# Technetium-99m stannous phytate versus technetium-99m sulfur colloid for sentinel lymph node mapping in breast cancer: A single-center retrospective study

**DOI:** 10.1097/MD.0000000000050258

**Published:** 2026-08-28

**Authors:** Chunhuai Liao, Jian Lu, Luz Angela Torres-de la Roche, Rudy Leon De Wilde, Tao Liu, Wenjie Shi, Qiang Wang, Yicheng Jiang, Rui Zhuo, Maya Sophie de Wilde

**Affiliations:** a Carl von Ossietzky University Oldenburg, Oldenburg, Germany; b Department of Breast Surgery, Guilin Municipal Hospital of Traditional Chinese Medicine, Guilin, China; c Department of General Medicine, 924th Hospital of the Joint Logistics Support Force of the Chinese People’s Liberation Army, Guilin, China; d University Hospital for Gynecology, Pius Hospital, University Medicine Oldenburg, Oldenburg, Germany; e Department of Nuclear Medicine, Guilin Hospital of Integrated Traditional Chinese and Western Medicine, Guilin, China.

**Keywords:** breast cancer, lymphoscintigraphy, sentinel lymph node imaging, technetium-99m stannous phytate, technetium-99m sulfur colloid

## Abstract

This study compares technetium-99m stannous phytate (99mTc-PHY) plus methylene blue versus technetium-99m sulfur colloid (99mTc-SC) plus methylene blue for sentinel lymph node (SLN) imaging in early-stage breast cancer (BC). We retrospectively reviewed early-stage BC patients treated from 2019 to 2024. All underwent sentinel lymph node biopsy using either 99mTc-PHY with methylene blue or 99mTc-SC with methylene blue as tracers. A Neoprobe 2000r detector was used to locate nodes. A total of 1151 patients were enrolled in the study, of whom 669 received 99mTc-SC and 482 received 99mTc-PHY (mean age 52 years). The SLN detection rates were 98.8% and 97.3%, respectively (*P* = .06). The positive SLN rates for metastasis (14.8% vs 11.7%; *P* = .13) and the median SLN counts were comparable (4 vs 3; *P* = .27). In multivariable analysis, higher tumor stage (T1–T2) but not histological type (ductal or lobular) was independently associated with increased SLN count in both groups (T1: incidence rate ratios = 1.318; T2: incidence rate ratios = 1.686; both *P* < .001). The rate of axillary lymph node dissection was found to be significantly lower in the 99mTc-SC group (26.5% vs 43.6%; *P* = .03). However, the reasons for this difference were not identified. No perioperative adverse events were reported. The present findings provide support for the use of 99mTc-PHY as a feasible tracer in the context of SLN detection in BC patients, thus exhibiting no statistically significant difference in detection rates compared to 99mTc-SC.

## 1. Introduction

Breast cancer (BC) is the most common malignancy and a leading cause of cancer death among women worldwide.^[1]^ Despite substantial advances in early screening and standardized therapeutic strategies, approximately 2.3 million women are newly diagnosed with BC globally each year.^[2]^ Axillary lymph node (ALN) status is a critical prognostic indicator and a core basis for determining tumor stage and individualized treatment regimens for BC patients. Axillary lymph node dissection (ALND) has historically been the standard for evaluating ALN involvement, but it frequently causes upper limb numbness, persistent pain, and lymphedema, which significantly impair long-term quality of life.^[3]^ To avoid these complications, sentinel lymph node biopsy (SLNB) has been widely popularized as a minimally invasive alternative for evaluating axillary metastatic status in clinically node-negative BC patients.^[4]^

Sentinel lymph node (SLN) refers to the first lymph node that receives lymphatic drainage and tumor cell metastasis from the primary breast tumor. SLNB enables accurate histopathological assessment of regional lymph node metastatic status via intraoperative localization and biopsy of SLNs, and has gradually replaced traditional ALND for axillary staging of BC in global clinical practice.^[5]^ The American Society of Clinical Oncology guidelines currently recommend SLNB as the first-line modality for ALN staging in BC patients,^[6]^ which is particularly advantageous for patients with additional sentinel lymphatic drainage to extra-axillary nodal basins such as the internal mammary region.^[7,8]^ Radionuclide tracers are essential for SLN identification and accurate SLNB assessment.^[9]^ At present, the combined application of radioactive isotopes and blue dye is recognized as the gold standard for SLN localization, owing to its superior SLN identification rate and minimal false-negative rate.^[10]^

Technetium-99m sulfur colloid (99mTc-SC) is the most established and widely applied radiotracer for SLN mapping, and remains the preferred agent in European and American clinical practice.^[11,12]^ As a non-biological macromolecular polymer particle with a broad size distribution, it requires standardized filtration before use to obtain uniform micro-particles. After interstitial injection, 99mTc-SC is passively taken up by lymphatic vessels in a size-dependent manner and mechanically retained in lymph node sinuses and macrophages.^[13]^ This tracer exhibits features including stable lymph node retention, clear hierarchical imaging and high detection stability, with numerous clinical studies verifying its high SLN detection rate and diagnostic accuracy.^[14,15]^ It has also been approved as a commercial lymphatic imaging agent in Europe. The main drawbacks are its nonuniform particle size distribution and the complex preoperative preparation, which can affect the repeatability and stability of imaging results.

Technetium-99m stannous phytate (99mTc-PHY) is another radiotracer for SLN imaging with distinct physicochemical characteristics. It is a small-molecule compound without inherent particulate properties. After interstitial injection, it binds calcium ions in tissue and lymphatic fluid to form in situ colloidal microparticles, which are subsequently phagocytosed by reticuloendothelial cells in lymph nodes, enabling lymphatic imaging. This tracer offers several clinical advantages, including simple preparation and good stability. Compared with 99mTc-SC, it is known to have shorter lymph node retention and slower lymphatic migration. Initially, 99mTc-PHY was introduced into nuclear medicine by Subramanian for evaluating reticuloendothelial cell activity in the liver and bone marrow. Subsequent studies have confirmed its value in assessing liver function and stratifying the severity of liver injury,^[16,17]^ and the in situ formed 99mTc-PHY nanocolloids display similar lymph node biodistribution characteristics to the reticuloendothelial system, enabling dynamic visualization of lymph node involvement and lymphatic drainage pathways via lymphoscintigraphy.^[18]^

Although 99mTc-SC is a classic tracer, the process of preparing it is relatively complicated, and the heterogeneous particle size may affect the stability and reproducibility of the results. Therefore, a simpler and more stable alternative would be clinically useful. However, published head-to-head comparisons between 99mTc-SC and 99mTc-PHY have not reached a consistent conclusion. Some studies favor 99mTc-SC for SLN detection, whereas others report that 99mTc-PHY achieves equivalent or even higher detection rates with easier handling. For example, Nomori et al found a significantly higher detection rate for 99mTc-PHY than tin colloid (89% vs 74%) in lung cancer patients.^[19]^ This inconsistency leaves room for further evaluation of 99mTc-PHY in SLN imaging.

Given its simple preparation, potentially high detection rate, and distinct mechanism, 99mTc-PHY is a promising tracer for SLN localization. The study aimed to evaluate, through a retrospective analysis, the tracing efficacy of 99mTc-PHY versus 99mTc-SC in patients with early-stage BC, specifically regarding SLN localization and detection rate by lymphoscintigraphy.

## 2. Methods

### 2.1. Study design and population

A retrospective single-center comparative study was conducted at the Department of Breast Medicine, Guilin Municipal Hospital of Traditional Chinese Medicine (Approval No. 2025-ky-lw-010-02), including patients treated between 2019 and 2024. 99mTc-SC was used for SLN mapping from 2019 to 2021, and 99mTc-PHY has been routinely used since 2022. Inclusion criteria were: age ≥ 18 years, histologically confirmed BC (clinical stage Tis N0M0, T1-T4N0M0) and scheduled for BC surgery including SLNB. Exclusion criteria were: prior ipsilateral axillary surgery, confirmed ALN metastasis (by clinical examination, imaging, or fine-needle aspiration cytopathology), previous breast radiotherapy, inflammatory BC, pregnancy, or lactation. The data presented herein is exclusively from patients with early-stage BC (stage Tis-T2 N0M0).

### 2.2. Materials and equipment

The Neoprobe 2000r detector. Single-photon emission and X-ray computed tomography scanner, model: Symbia Intevo Bold. Sodium stannous phytate for injection comes from Jiangyuan Pharmaceutical Factory of Jiangyuan Industrial Technology and Trade Co., Ltd. in Wuxi City, Jiangsu Province. Methylene blue comes from China Jichuan Pharmaceutical Group Co., Ltd.

### 2.3. Preoperative lymphatic scintigraphy

**Figure 1.**
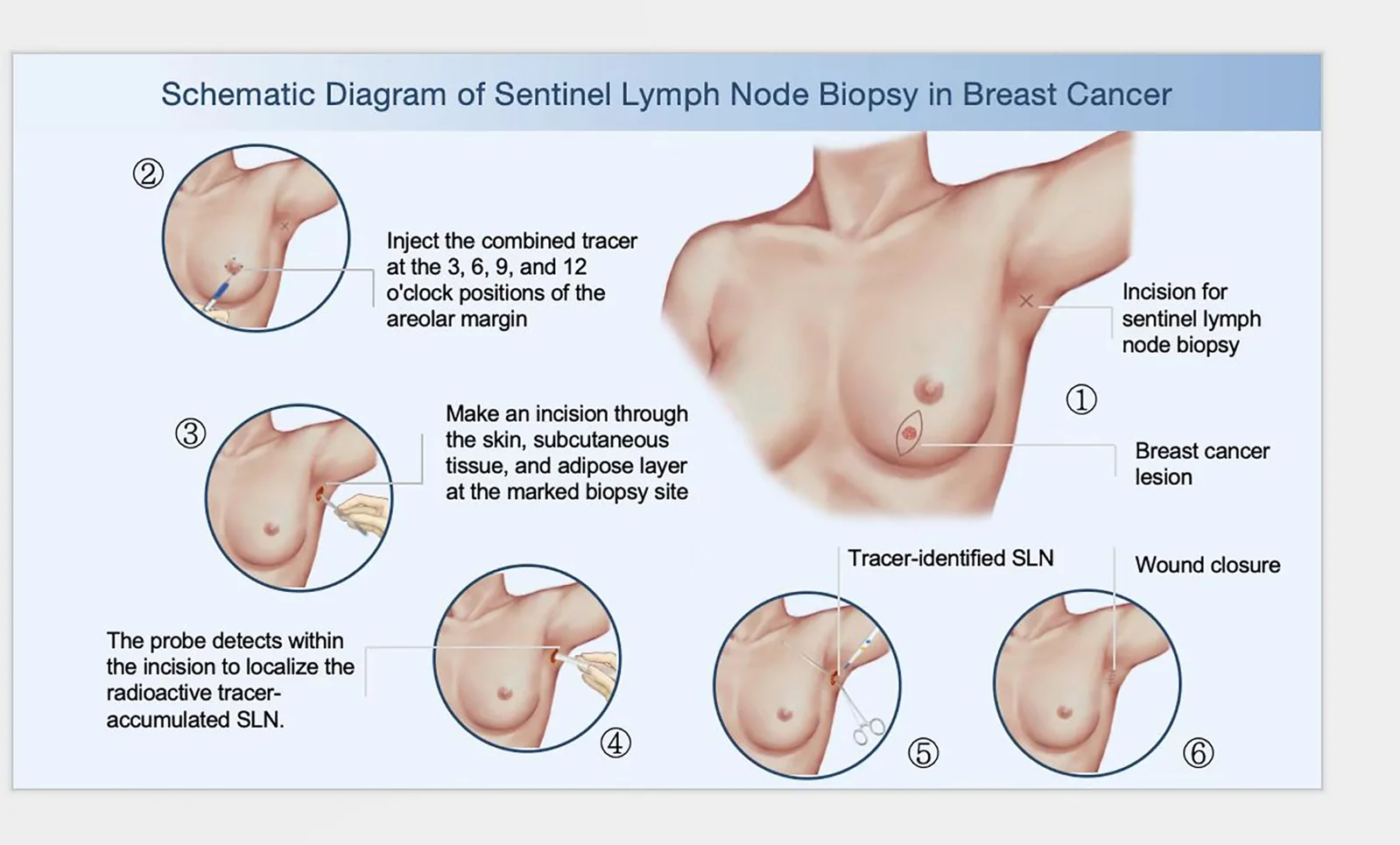
Schematic diagram of sentinel lymph node biopsy in breast cancer. This figure shows the steps of sentinel lymph node biopsy for breast cancer, including tumor location, tracer injection site and method, surgical incision selection, R-detector use, sentinel lymph node resection, and postoperative incision closure. SLN = sentinel lymph node.

Preoperatively, 0.5 mCi/0.1 mL of either 99mTc-PHY or 99mTc-SC was injected subcutaneously at the 3, 6, 9, and 12 o’clock positions around the areola, the primary tumor, or the post-biopsy residual cavity wall, followed by local massage for 2 to 3 minutes. Patients were observed for 30 minutes after tracer injection. The SLN was defined as the radioactive hotspot closest to the injection site, displaying the earliest appearance and the highest nuclide concentration. This hotspot was repeatedly localized with a handheld gamma probe, and the skin surface was marked with a marker when the point source coincided with the area of maximal radioactivity. Intraoperatively, 2 mL of 1% methylene blue was injected subcutaneously at the 3, 6, 9, and 12 o’clock positions around the areola or the primary tumor. SLNB was performed 10 to 15 minutes after tracer injection. ALNs with radioactive uptake were identified using a handheld gamma probe. All blue-stained and/or radioactive SLNs, as well as those with radioactivity exceeding 10% of the highest count rate, were harvested. The harvested SLNs were submitted for intraoperative pathological examination, including touch imprint cytology, frozen section and postoperative histopathological analysis (Fig. 1).

### 2.4. Statistical analysis

SLN detection rate was defined as the proportion of patients in whom at least one SLN was successfully identified and harvested during surgery. Adverse events were graded per CTCAE 5.0 as mild, moderate, or severe. Events occurring before surgery were defined as tracer-related. Postoperative complication assessments were performed by a nurse at 15 and 30 minutes, followed by a 24-hour evaluation by ward nurses.

Normality and homogeneity of variance tests were performed for continuous data and were presented as mean ± standard deviation or median (interquartile range [IQR]) and compared using the independent-samples *t*-test or Mann–Whitney *U* test, as appropriate, with median differences estimated by the Hodges–Lehmann method. Categorical variables were expressed as counts (n) and percentages (%) and compared using the χ^2^ test or Fisher exact test. A multivariate negative binomial regression model was used to assess factors associated with the number of retrieved SLNs, with results reported as incidence rate ratios (IRR) and 95% confidence interval (CI). All tests were two-sided, and *P* < .05 was considered statistically significant. Analyses were performed using SPSS 27.0.

## 3. Results

**Figure 2.**
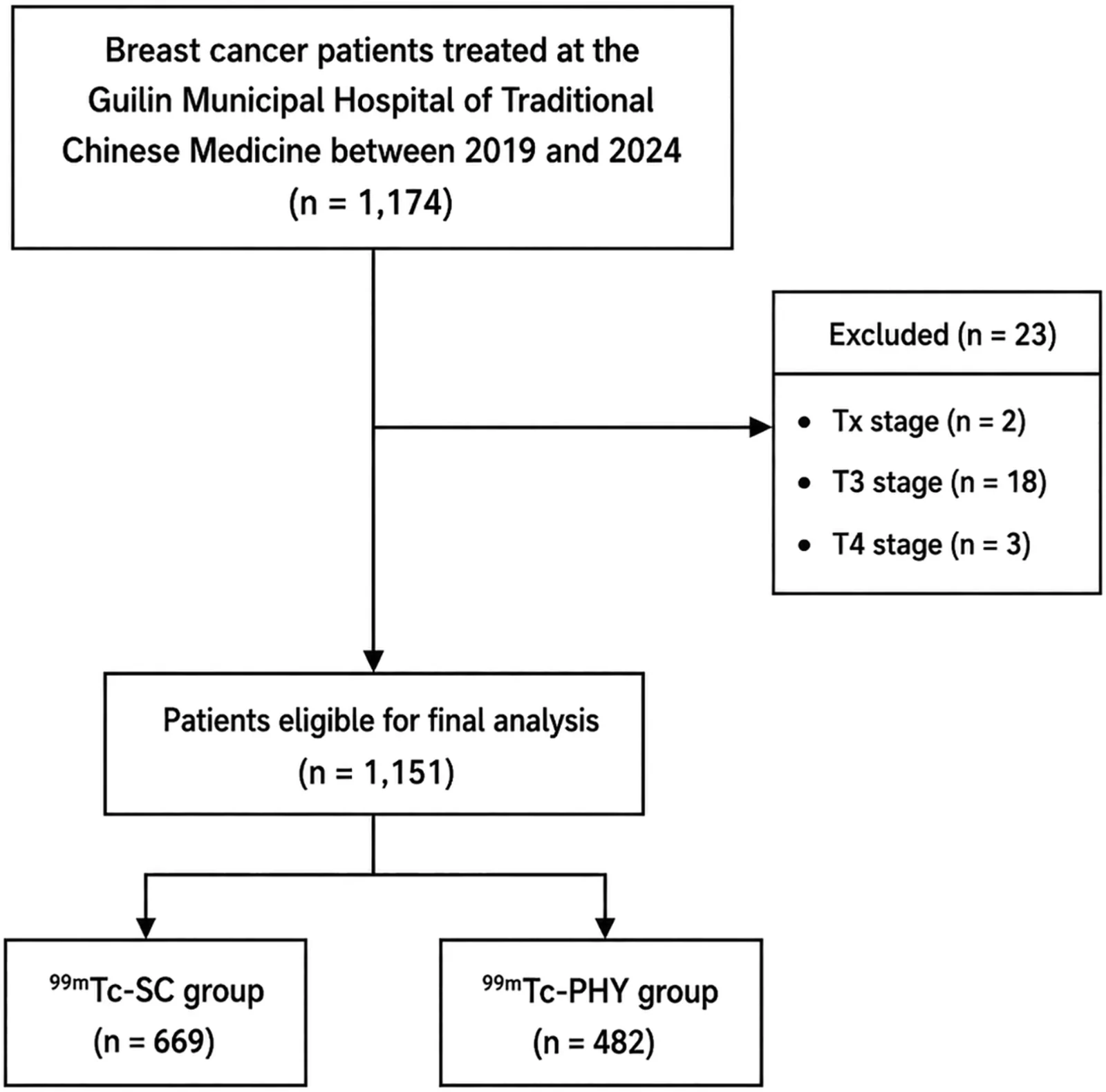
Patient selection flowchart. A total of 1174 breast cancer patients treated at the Guilin Municipal Hospital of Traditional Chinese Medicine from 2019 to 2024 were initially screened. After excluding 23 patients (Tx, n = 2; T3, n = 18; T4, n = 3), 1151 patients were included in the final analysis. Among them, 669 received 99mTc-SC and 482 received 99mTc-PHY. 99mTc-PHY = technetium-99m phytate, 99mTc-SC = technetium-99m sulfur colloid.

1151 patients were included in this retrospective analysis, with 669 patients in the 99mTc-SC group and 482 patients in the 99mTc-PHY group (Fig. 2). No missing data were present for any of the key study variables.

No significant differences were observed in age (52.66 ± 11.13 vs 52.35 ± 10.53 years, *P* = .68), sex distribution (*P* = .51), menopausal status (*P* = .73), affected breast side (*P* = .41), body mass index (23.64 ± 3.32 vs 23.67 ± 3.08 kg/m^2^, *P* = .68), tumor stage (*P* = .21), histological type (*P* = .09), tumor location (*P* = .65), or surgical procedure (*P* = .10; Table 1).

**Table 1:** Clinical characteristics of patients.

	99mTc-SC	99mTc-PHY	*P* value
Age (yrs Mean ± SD)	52.66 ± 11.13	52.35 ± 10.53	.68
Gender	669	482	.51
Female	667 (99.7%)	482 (100%)	
Male	2 (0.3%)	0 (0.0%)	
Menopausal status			.73
Premenopausal	322 (48.1%)	237 (49.2%)	
Postmenopausal	347 (51.9%)	245 (50.8%)	
Affected breast			.41
Left	339 (50.7%)	256 (53.1%)	
Right	330 (49.3%)	226 (46.9%)	
BMI (kg/m^2^, Mean ± SD)	23.64 ± 3.32	23.67 ± 3.08	.68
Tumor stage			.21
Tis	98 (14.7%)	55 (11.4%)	
T1	304 (45.4%)	237 (49.2%)	
T2	267 (39.9%)	190 (39.4%)	
Histological type			.09
Ductal carcinoma	497 (74.3%)	384 (79.7%)	
Lobular carcinoma	27 (4.0%)	17 (3.5%)	
Other	145 (21.7%)	81 (16.8%)	
Tumor location			.65
Upper outer quadrant	321 (48.0%)	225 (46.7%)	
Lower outer quadrant	61 (9.1%)	52 (10.8%)	
Upper inner quadrant	155 (23.2%)	118 (24.5%)	
Lower inner quadrant	86 (12.8%)	51 (10.6%)	
Central region	46 (6.9%)	36 (7.4%)	
Surgical procedure			.10
Mastectomy	287 (42.9%)	236 (49.0%)	
Breast-conserving surgery	346 (51.7%)	227 (47.1%)	
Breast reconstruction surgery	36 (5.4%)	19 (3.9%)	

Data are presented as mean ± standard deviation (SD) for continuous variables and as number (percentage) for categorical variables. Baseline demographic, clinicopathological, and surgical characteristics were compared between patients who underwent sentinel lymph node biopsy using 99mTc-SC and those who underwent the procedure using 99mTc-PHY. Continuous variables were compared using the independent-samples *t* test, and categorical variables were compared using the chi-square test or Fisher exact test, as appropriate. All *P* values are two-sided.

99mTc-PHY = technetium-99m stannous phytate, 99mTc-SC = technetium-99m sulfur colloid, BMI = body mass index, SD = standard deviation.

The SLN detection rate (Table 2) was 98.8% (661/669) for 99mTc-SC and 97.3% (469/482) for 99mTc-PHY, with no statistically significant difference (*P* = .06). SLN identification failed in 8 patients (1.2%) in the 99mTc-SC group and 13 (2.7%) in the 99mTc-PHY group. Given the low failure rate, no subgroup or additional baseline analysis was performed. Among patients with successful SLN identification, the SLN positivity rate (defined as metastasis confirmed on frozen section biopsy) was also comparable between the 2 groups (14.8% vs 11.7%; *P* = .13). Among SLN positive patients, the rate of completion ALND was significantly lower in the 99mTc-SC group (26.5% vs 43.6%; *P* = .03).

**Table 2:** Comparison of patient-level sentinel lymph node detection and related outcomes between the Technetium-99m sulfur colloid and Technetium-99m stannous phytate groups.

Outcome	99mTc-SC (n = 669)	99mTc-PHY (n = 482)	*P* value
SLN detection rate	661/669 (98.8%)	469/482 (97.3%)	.06
SLN positivity rate	98/661 (14.8%)	55/469 (11.7%)	.13
ALND rate in SLN-positive	26/98 (26.5%)	24/55 (43.6%)	.03

Data are presented as number/total number (percentage). SLN detection rate was defined as the proportion of patients in whom at least one SLN was successfully identified. SLN positivity rate was calculated among patients with successful SLN identification. The ALND rate was calculated among patients with positive SLNs. Comparisons between groups were performed using the chi-square test or Fisher exact test, as appropriate. All *P* values are two-sided.

99mTc-PHY = technetium-99m stannous phytate, 99mTc-SC = technetium-99m sulfur colloid, ALND = axillary lymph node dissection, SLN = sentinel lymph node.

The median number of detected SLNs (Table 3) was 4 (IQR 3–4) in the 99mTc-SC group (n = 661) and 3 (IQR 2–5) in the 99mTc-PHY group (n = 469). The median difference was 0 (95% CI: −1 to 1), with no significant difference (*P* = .27).

**Table 3:** Comparison of retrieved sentinel lymph node counts between the technetium-99m sulfur colloid and Technetium-99m stannous phytate groups.

Group	N	Median (P_25_, P_75_)	Median difference (SC − PHY)	95% CI	*P* value
99mTc-SC	661	4 (3, 4)	–	–	–
99mTc-PHY	469	3 (2, 5)	–	–	–
99mTc-SC vs 99mTc-PHY	–	–	0	(−1 to 1)	.27

Data are presented as median (25th, 75th percentiles). The median difference (SC minus PHY) and its 95% confidence interval were estimated using the Hodges–Lehmann method. The *P* value was calculated using the Wilcoxon rank-sum (Mann–Whitney) test.

95% CI = 95% confidence interval, 99mTc-PHY = technetium-99m stannous phytate, 99mTc-SC = technetium-99m sulfur colloid.

As shown in Table 4, in the 99mTc-SC group, the most frequent node count was 4 (29.9%), followed by 3 (26.6%) and 2 (18.6%). In the 99mTc-PHY group, the distribution was more dispersed: 3 (24.3%), 4 (21.5%), 5 (20.5%), and 2 (19.8%). The 99mTc-PHY group showed a slightly wider distribution of retrieved SLN counts, including a higher proportion of patients with 1 node (8.5% vs 3.6%).

**Table 4:** Distribution of the number of detected sentinel lymph nodes in the 2 groups.

Number of detected SLNs	99mTc-SC (n = 661) n (%)	99mTc-PHY (n = 469) n (%)
1	24 (3.6)	40 (8.5)
2	123 (18.6)	93 (19.8)
3	176 (26.6)	114 (24.3)
4	198 (29.9)	101 (21.5)
5	105 (15.9)	96 (20.5)
6	27 (4.1)	18 (3.8)
7	8 (1.2)	7 (1.5)

Data are presented as number of patients (%), with percentages calculated using the group-specific total of patients with successful SLN detection as the denominator (99mTc-SC, n = 661; 99mTc-PHY, n = 469).

99mTc-PHY = technetium-99m stannous phytate, 99mTc-SC = technetium-99m sulfur colloid, SLNs = sentinel lymph nodes.

In multivariable regression analysis (Table 5), no significant difference in SLN count was observed between the 99mTc-PHY and 99mTc-SC groups (IRR = 1.028, 95% CI: 0.942–1.123; *P* = .54). Compared with Tis, higher tumor stages were independently associated with SLN count, with progressively increased counts in T1 (IRR = 1.318, 95% CI: 1.106–1.570; *P < *.001) and T2 (IRR = 1.686, 95% CI: 1.414–2.012; *P* < .001). No significant differences were identified for histological subtype, including invasive ductal carcinoma (IRR = 0.954, 95% CI: 0.867–1.050; *P = *.33) and invasive lobular carcinoma (IRR = 1.082, 95% CI: 0.925–1.266; *P* = .32). No adverse events were noted in either the 99mTc-PHY or 99mTc-SC group intraoperatively or within 24 hours postoperatively.

**Table 5:** Multivariable negative binomial regression of retrieved sentinel lymph node count.

Variable	IRR	95% CI	*P* value
99mTc-PHY vs 99mTc-SC	1.028	0.942–1.123	.54
T stage			
T1	1.318	1.106–1.570	<.001
T2	1.686	1.414–2.012	<.001
Histological type			
Invasive ductal carcinoma	0.954	0.867–1.050	.33
Invasive lobular carcinoma	1.082	0.925–1.266	.32

IRRs and 95% CIs were estimated using a multivariable negative binomial regression model. Covariates included tracer type (99mTc-PHY vs 99mTc-SC), T stage (T1 and T2 vs Tis), and histological type (invasive ductal carcinoma and invasive lobular carcinoma vs other histological types). Reference categories were 99mTc-SC, Tis, and other histological types.

95% CI = 95% confidence interval, 99mTc-PHY = technetium-99m stannous phytate, 99mTc-SC = technetium-99m sulfur colloid, IRR = incidence rate ratios.

## 4. Discussion

SLNB has been adopted widely during BC staging for detecting regional metastasis^[6]^ and it is recommended by the American Society of Clinical Oncology as the first-line method for ALN staging.^[7]^ This approach is particularly valuable for BC patients who also have sentinel lymphatic drainage to other nodal basins, such as the internal mammary region. Previous studies have shown that several radiopharmaceuticals can be used for SLN detection, including 99mTc-hydroxyethyl starch, 99mTc-dextran, 99mTc-human serum albumin, 99mTc-sulfur colloid, 99mTc-antimony trisulfide colloid, and 99mTc-albumin colloid.^[20–22]^ However, the optimal tracer for SLN mapping has not yet been clearly established. 99mTc-PHY has been used as a tracer for SLN in BC patients, but the evidence supporting its efficacy in this setting remains limited. Most of the available data come from other solid tumors. Whether a similar advantage holds for 99mTc-PHY over 99mTc-SC in BC has not been directly examined. We therefore compared 99mTc-PHY with 99mTc-SC directly in the present cohort. The results demonstrated similar detection rates (98.8% for 99mTc-SC and 97.3% for 99mTc-PHY) are consistent with the performance reported in the SLNB literature. A large meta-analysis by Niebling et al reported pooled SLN identification rates of 94% for radiocolloid alone and 95% for radiocolloid combined with blue dye in BC patients, showing that both agents in the present study fall well within acceptable clinical ranges.^[23]^

The detection rate of 99mTc-PHY in this study was found to be marginally lower than that of 99mTc-SC, though this difference was not statistically significant (*P* = .06). This finding are consistent with the results of a multi-institutional Iranian study, which reported a detection rate of 91.8% for 99mTc-PHY, which additionally noted that the performance of 99mTc-PHY was comparable to that of 99mTc-SC, despite the former being more readily producible locally.^[24]^ Moreover, in a randomized controlled study directly comparing the 2 tracers, Yang et al^[25]^ found no significant difference in the number of identified SLNs or in accuracy metrics between 99mTc-PHY and 99mTc-SC, consistent with the present findings. In this broader context, both tracers appear clinically interchangeable with respect to the binary outcome of successful localization.

Small differences in SLN retrieval patterns between 99mTc-PHY and 99mTc-SC were observed, though these differences are not possible to explain based on tumor size or histological type. In the 99mTc-PHY group, the distribution of retrieved nodes was more dispersed, with a higher proportion of patients having either only 1 or 5 SLNs compared with the 99mTc-SC group. By contrast, the 99mTc-SC group displayed a more concentrated distribution centered around 4 nodes. Whether differences in SLN retrieval patterns contributed to the detection rate cannot be determined from the present data, and the observed difference should be interpreted cautiously given the retrospective design and potential influence of unmeasured clinical factors.

Among patients with successful SLN identification, the positivity (metastasis) rate was similar between the 2 tracer groups (14.8% vs 11.7%, *P* = .13), though there was a statistically significantly lower ALND rate in the 99mTc-SC group (*P* = .03). The observed variations could be attributed to temporal changes in axillary management protocols, patient selection criteria, surgeon preference, and institutional practice patterns. Consequently, it is not possible to infer a relationship between tracer type and ALND indication from the data presented. Furthermore, the present study was not designed to evaluate their impact on intraoperative axillary management decisions. The clinical criteria for omitting ALND in SLN-positive patients have shifted over time. In the context of contemporary axillary management, reliable SLN mapping remains essential for identifying patients who may safely avoid ALND, provided that current guideline criteria are met.^[26]^ The ACOSOG Z0011 trial^[27]^ demonstrated that in patients with T1–T2 BC undergoing breast-conserving surgery and with 1 or 2 positive SLNs, omitting ALND did not compromise 10-year overall survival. Other studies^[28–31]^ have extended this principle to selected patients with low-volume nodal disease (micrometastases or isolated tumor cells). In the present study, all patients with negative SLNs successfully avoided ALND, highlighting the crucial role of SLNB in preventing unnecessary surgical procedures and related complications.

An ideal SLN tracer should improve the diagnostic accuracy of SLNB and reduce the false-negative rate, thereby better supporting clinical decision-making. Particle size critically determines lymphatic distribution velocity and retention time: larger particles tend to migrate more slowly and remain longer in the node.^[32]^ Unfiltered 99mTc-SC exhibits a broad particle size distribution ranging from 15 to > 5000 nm, with a mean diameter of 305 to 340 nm.^[33]^ In routine clinical practice, filtration through a 0.22-μm membrane removes large aggregates, resulting in a filtered preparation with most particles between 100 and 220 nm, which aligns with the optimal 100 to 200 nm window for SLN mapping, balancing rapid lymphatic transit and sufficient nodal retention.^[13]^ In contrast, 99mTc-PHY is not a preformed stable colloid but undergoes in vivo transformation after administration, as upon injection it interacts with endogenous calcium ions to form calcium phytate colloids, with the resulting particle size depending on calcium concentration and local physicochemical conditions.^[34]^ This calcium-dependent aggregation leads to an increase in effective particle size and may partially overlap with sulfur colloid, contributing to similar lymphatic distribution characteristics in SLN imaging.^[18,35]^ 99mTc-SC particles are generally larger than freshly prepared 99mTc-PHY, although after vascular injection 99mTc-PHY rapidly binds to plasma calcium to form larger particles of a similar size range.^[36,37]^ Differences in the physicochemical properties of the 2 tracers might explain the observed differences in SLN retrieval patterns. However, the broader distribution of retrieved SLN counts observed in the 99mTc-PHY group may also be explained by differences in lymphatic migration and nodal retention characteristics, although these parameters were not directly evaluated in the present study. The absence of difference in detection rate between the tracers analyzed in the present study is consistent with previous studies reporting comparable performance of 99mTc-based tracers in SLN mapping in BC.^[38–40]^

Finally, the multivariable analysis demonstrated no independent association between tracer type and SLN counts (IRR = 1.028, 95% CI: 0.942–1.123; *P* = .54), indicating comparable performance between 99mTc-PHY and 99mTc-SC after adjustment for clinicopathological factors. In contrast, tumor stage was the only determinant of SLN count, with higher counts observed in T1 and T2 disease compared with Tis (IRR = 1.318 and 1.686, respectively; both *P* < .001), consistent with prior evidence that SLN count is influenced by BC stage.^[41]^ Histological subtype showed no significant effect.

These findings suggest that SLN detection is primarily driven by tumor-related factors rather than radiotracer selection.

### 4.1. Limitations and strengths

The retrospective design of the study inherently limited the interpretation of the results. In addition, the analysis did not include all potential confounding factors, such as the site of injection, the time between injection and tracer testing, and the surgeon’s experience in lymph node dissection.^[42]^ Furthermore, as not all patients underwent histopathological examination of their ALNs, it was not possible to calculate the sensitivity and specificity values of the tracers. However, the relatively large sample size and low detection failure rates strengthen the main findings. Baseline demographic, clinical and pathological characteristics were comparable between groups, and multivariable negative binomial regression analysis was used to adjust for clinical confounders.

## 5. Conclusion

The present study shows that 99mTc-PHY is a safe SLN tracer in patients with early-stage BC and has exhibited no statistically significant difference in detection rates when compared with 99mTc-SC. In comparison with in situ disease, an increased number of nodes retrieved was found to be statistically associated with advanced tumor stages (T1–T2). Further comparative studies could establish the clinical value and the feasibility of 99mTc-PHY as a routine tracer for BC staging.

## Author contributions

**Conceptualization:** Rudy Leon De Wilde, Rui Zhuo.

**Data curation:** Qiang Wang.

**Formal analysis:** Yicheng Jiang, Chunhuai Liao.

**Investigation:** Jian Lu, Tao Liu, Qiang Wang.

**Methodology:** Chunhuai Liao, Jian Lu, Luz Angela Torres-de la Roche.

**Project administration:** Rudy Leon De Wilde, Rui Zhuo.

**Resources:** Rudy Leon De Wilde, Rui Zhuo.

**Software:** Qiang Wang, Yicheng Jiang.

**Supervision:** Wenjie Shi.

**Validation:** Wenjie Shi, Yicheng Jiang, Maya Sophie de Wilde.

**Writing – original draft:** Chunhuai Liao, Jian Lu, Luz Angela Torres-de la Roche.

**Writing – review & editing:** Chunhuai Liao, Jian Lu, Luz Angela Torres-de la Roche, Maya Sophie de Wilde.
